# THE ASSOCIATION OF NON-SEVERE COVID-19 INFECTION AND PROGRESSION TO FRAILTY AMONG ROBUST OLDER VETERANS

**DOI:** 10.1093/geroni/igad104.3628

**Published:** 2023-12-21

**Authors:** Natasha Melo Resendes, Fei Tang, Dominique Tosi, Iriana Hammel

**Affiliations:** Miami VA Healthcare System, Miami, Florida, United States; Miami VA Healthcare System, Miami, Florida, United States; Miami VA Healthcare System, Miami, Florida, United States; Miami VA Healthcare System, Miami, Florida, United States

## Abstract

**Background:**

Frailty is a multisystem syndrome characterized by vulnerability to stressors, such as the infection with COVID-19. We set out to examine if mild COVID-19 infection impacted frailty onset among older robust veterans, and whether other factors were associated with developing frailty.

**Methods:**

Retrospective cohort study of U.S. Veterans 55 years and older who had a SARS-CoV-2 infection between 3-15-2020, and 11-30-2020, were active VHA users/and had a VA Frailty Index (VA-FI) of 0.0 at time of diagnosis. We used nationwide VHA data from the VA COVID-19 Shared Data Resource database. We excluded Veterans hospitalized after contracting COVID-19, and who died within the 25-month follow-up. Cox proportional hazard model was used to assess the association between COVID-19 infection and developing frailty or (pre-frailty + frailty), after adjusting for age, BMI, race, ethnicity, gender, smoking, and rurality.

**Results:**

86061 Veterans aged 55 and older met inclusion criteria. Mean age was 68.4 yrs (SD=7.8) with 91.0% (78347) males. 7561 (8.8%) tested positive for COVID-19, and 78500 (91.2%) tested negative. The COVID-19 positive group had a higher percentage of black and city-dwelling Veterans than the COVID-19 negative group. After adjustments listed above, testing positive for COVID-19 was associated with an increased hazard of becoming frail (adjusted HR=1.63, 95%CI: 1.24-2.13), and pre-frail + frail (adjusted HR=1.77, 95%CI: 1.53-2.06).

**Conclusion:**

We showed that mild COVID-19 was associated with higher incidence of frailty and pre-frailty. Follow-up post COVID-19 infection should include a comprehensive geriatric assessment for early frailty identification and intervention.

